# *Neisseria meningitidis*-Induced Caspase-1 Activation in Human Innate Immune Cells Is LOS-Dependent

**DOI:** 10.1155/2019/6193186

**Published:** 2019-05-06

**Authors:** Berhane Asfaw Idosa, Anne Kelly, Susanne Jacobsson, Isak Demirel, Hans Fredlund, Eva Särndahl, Alexander Persson

**Affiliations:** ^1^iRiSC-Inflammatory Response and Infection Susceptibility Centre, Faculty of Medicine and Health, Örebro University, SE-701 82 Örebro, Sweden; ^2^School of Medical Sciences, Faculty of Medicine and Health, Örebro University, SE-701 82 Örebro, Sweden; ^3^Karolinska University Hospital, Solna, SE-171 76 Stockholm, Sweden; ^4^Department of Laboratory Medicine, Faculty of Medicine and Health, Örebro University, SE-701 82 Örebro, Sweden

## Abstract

Meningococcal disease such as sepsis and meningitidis is hallmarked by an excessive inflammatory response. The causative agent, *Neisseria meningitidis*, expresses the endotoxin lipooligosaccharide (LOS) that is responsible for activation of immune cells and the release of proinflammatory cytokines. One of the most potent proinflammatory cytokines, interleukin-1*β* (IL-1*β*), is activated following caspase-1 activity in the intracellular multiprotein complex called inflammasome. Inflammasomes are activated by a number of microbial factors as well as danger molecules by a two-step mechanism—priming and licensing of inflammasome activation—but there are no data available regarding a role for inflammasome activation in meningococcal disease. The aim of this study was to investigate if *N. meningitidis* activates the inflammasome and, if so, the role of bacterial LOS in this activation. Cells were subjected to *N. meningitidis*, both wild-type (FAM20) and its LOS-deficient mutant (*lpxA*), and priming as well as licensing of inflammasome activation was investigated. The wild-type LOS-expressing parental FAM20 serogroup C *N. meningitidis* (FAM20) strain significantly enhanced the caspase-1 activity in human neutrophils and monocytes, whereas *lpxA* was unable to induce caspase-1 activity as well as to induce IL-1*β* release. While the *lpxA* mutant induced a priming response, measured as increased expression of *NLRP3* and *IL1B*, the LOS-expressing FAM20 further increased this priming. We conclude that although non-LOS components of *N. meningitidis* contribute to the priming of the inflammasome activity, LOS *per se* is to be considered as the central component of *N. meningitidis* virulence, responsible for both priming and licensing of inflammasome activation.

## 1. Introduction

Meningococci are considered commensals in the nasopharyngeal mucosa, and it is estimated that 10-40% of the general population are asymptomatic carriers, providing the reservoir for this obligate human pathogen [1, 2]. However, the interconnection between asymptomatic carriage and disease development is not known. *Neisseria meningitidis* is the major causative agent of sepsis and bacterial meningitis globally [3] with the highest incidence of meningococcal disease occurring in the meningitis belt of sub-Saharan Africa that extends across the regions from Senegal in the west to Ethiopia in the east [3, 4]. The mortality rate ranges from 4 to 6% in the case of meningitis up to 40% in the case of severe sepsis and septic shock [5]. Severity of meningococcal disease varies among individuals, in whom some suffer from meningitis alone, while others may develop meningococcemia with mild or severe sepsis [6, 7]. It is generally believed that the bacterial virulence factors in combination with the individual's unique immune system determine disease severity.

The virulence of *N. meningitidis* is influenced by several factors including capsular polysaccharides, expression of type IV pili (Tfp), outer membrane proteins (Opa and Opc), porins (PorA and PorB), and lipooligosaccharide (LOS). The dramatic onset of meningococcal disease is reported to rely on extensive release of LOS from the outer membrane of *N. meningitidis*, causing immune cell activation and release of proinflammatory cytokines [8]. Further, endotoxic shock, characterized by an amplified or harmful response of the host immune system, is mediated by LOS during meningococcal infection and the severity of meningococcal disease correlates with the level of LOS circulating in the bloodstream as it influences the intensity of the immune response [9, 10]. The clinical feature of infection by pathogenic *N. meningitidis* is a host innate immune-driven inflammatory response, characterized by a potent neutrophil influx. The involvement of neutrophils in meningococcal disease is debated [11], but neutropenia is found associated with poor prognosis [12].

Cells of the innate immune system express pattern recognition receptors (PRRs), which specifically sense microbial structures (PAMPS, pathogen-associated molecular patterns), such as peptidoglycan, lipopolysaccharide (LPS), and microbial nucleic acids [13, 14]. The PRRs include Toll-like receptors (TLRs), which are found on the cell surface, and intracellular nucleotide-binding domain leucine-rich repeat containing receptors (NLRs) that detect pathogens in the cytosol. Upon activation, some NLRs initiate the formation of cytoplasmic multiprotein complexes called inflammasomes that facilitate the activation of caspase-1, which results in the maturation of the pro-forms of interleukin-1*β* (IL-1*β*) and IL-18 into biologically active cytokines [14, 15]. Meningococcal disease is hallmarked by elevated concentrations of proinflammatory cytokines and chemokines, including IL-1*β*, tumor necrosis factor (TNF), IL-6, and CXCL8 (formerly known as IL-8) [16–18].

The NLR family pyrin domain-containing 3 (NLRP3) can be activated by a wide range of microbial stimuli [19, 20] to form the NLRP3 inflammasome that mediates IL-1*β* secretion in primary immune cells, including neutrophils and monocytes [21, 22]. Despite being relatively well characterized, no data regarding a potential role for inflammasome activation in meningococcal disease is currently available. The aim of the current study was to investigate if *N. meningitidis* mediates inflammasome activation and, if so, what role the meningococcal virulence factor LOS plays in this process.

## 2. Materials and Methods

### 2.1. Cells and Bacteria Preparation

Peripheral blood from healthy blood donors was collected in EDTA tubes at Örebro University Hospital. Neutrophils were isolated from whole blood by density gradient centrifugation on PolymorphPrep and LymphoPrep reagents (AXIS-SHIELD poC AS, Oslo, Norway), as previously described [23, 24]. Due to the lack of gene manipulation tools in human primary neutrophils, THP1 cells were used as a proof-of-concept-experimental model. THP1 cells, both wild-type and cells deficient in MyD88 (THP1-defMYD88), NLRP3 (THP1-defNLRP3), or caspase-1 (THP1-defCASP1) (Invivogen, San Diego, CA), were maintained at a cell density of 1 × 10^6^ cells/mL in RPMI 1640 supplemented with 10% FBS, 2 mM L-glutamine, 1 mM sodium pyruvate, 50 U/mL penicillin, 50 *μ*g/mL streptomycin (Thermo Fisher Scientific, Rockford, IL), 100 *μ*g/mL normocin, and for selection purposes, 200 *μ*g/mL zeocin, and 100 *μ*g/mL hygromycin (Invivogen, San Diego, CA) was added according to the manufacturer's recommendation. Experiments were conducted under antibiotic free conditions by washing the cells 3 times with RPMI 1640.

The wild-type parental FAM20 serogroup C *N. meningitidis* and its LOS-deficient *lpxA* mutant (kindly provided by Professor Ann-Beth Jonsson, Stockholm University, Sweden) [8] were grown on GC Agar (3.6% Difco GC Medium Base agar supplemented with 1% haemoglobin, 10% horse serum, and 1% IsoVitaleX) (BD Diagnostics, Sparks, MD) for 16-20 h at 37°C in 5% CO_2_ atmosphere, and colonies were harvested by a sterile swab and suspended in PBS. Bacterial density was determined by measuring the OD_600_ and used to infect whole blood, isolated neutrophils, or THP1 cells at an MOI of 10 bacteria per cell.

### 2.2. Caspase-1 Detection in Whole Blood

To mirror the events taking place during blood-borne infections, whole blood was collected from healthy blood donors and subjected to *N. meningitidis*. Neutrophil and monocyte concentration was determined using a haematology analyser (Sysmex Corporation, Kobe, Japan) and used to calculate the MOI. Peripheral blood (300 *μ*L) was stimulated in 5 mL tubes with FAM20 or *lpxA* (at an MOI of 10 : 1 based on total neutrophil and monocyte count) [25, 26] and simultaneously stained for caspase-1 activity with FAM-YVAD-FMK (FLICA; Immunochemistry Technologies, Bloomington, MN) for 2 h at 37°C, as previously described [25, 27]. LPS from *Escherichia coli* (50 ng/mL) (Sigma-Aldrich St. Louis, MO) in combination with 1 mM ATP (Sigma-Aldrich, St. Louis, MO; ATP was added for the last half-hour of incubation) was used as positive control for inflammasome activation. Erythrocytes were lysed using EasyLyse reagent (DakoCytomation, Glostrup, Denmark) at room temperature for 15 min, and blood cells were labelled with RPE-CY5-conjugated mouse anti-human CD45 (DakoCytomation, Glostrup, Denmark) and ECD-conjugated mouse anti-human CD14 (Beckman Coulter; Immunotec, Marseille, France) to differentiate leukocyte populations. Neutrophils and monocytes were separated based on side scatter and CD45 and CD14 gating [25]. Nonspecific binding was analyzed using an isotypic control IgG1 FITC/RPE/RPE-CY5 (DakoCytomation, Glostrup, Denmark) and was used in the gating strategy (data not shown). Caspase-1 activity was determined by flow cytometry (FC500 Beckman Coulter, Fullerton, CA) detecting FLICA fluorescence, and the MFI value for each sample was normalized to its corresponding unstimulated control to calculate the fold change.

### 2.3. Detection of Active Caspase-1 by Fluorometric Assay in Isolated Human Neutrophils

Isolated neutrophils were seeded (1 × 10^6^ cells) in a 96-well plate and preincubated with 50 *μ*M Ac-YVAD-AMC (Enzo Life Sciences, New York, NY) in RPMI-1640 containing 10% FBS for 1 h at 37°C and 5% CO_2_. The cells were then stimulated with FAM20 or *lpxA* at an MOI of 10 for 6 h. Cleavage of the substrate by caspase-1 was measured in a fluorescent plate reader at excitation/emission 340/440 nm (FLUOstar Optima, Ortenberg, Germany).

### 2.4. Detection of Active Caspase-1 by Flow Cytometry in Isolated Human Neutrophils

Isolated neutrophils were seeded (1 × 10^6^ cells) in a 96-well plate and stimulated with FAM20, *lpxA*, purified LOS 1 *μ*g/mL (extracted from meningococcal strain 44/76, serotype B, kindly provided by Dr. Lisbeth Meyer Naess, Norwegian Institute of Public Health, Norway) [28], *lpxA* + LOS, or FAM20 + TRL4 signalling blockers (anti hCD14-IgA 500 ng/mL, LPS-RS ultrapure 1 *μ*g/mL, and polymyxin B 10 *μ*g/mL, Invivogen, San Diego, CA) and simultaneously stained for caspase-1 activity with FLICA in RPMI-1640 containing 10% FBS for 2 h at 37°C and 5% CO_2_. Neutrophils were labelled with anti-CD66b-PE (Nordic BioSite, Täby, Sweden), and caspase-1 activity was determined by flow cytometry (Gallios Beckman Coulter, Fullerton, CA). In experiments designed to prime cells, isolated neutrophils were preincubated with the TLR1/2 agonist Pam3CSK4 (2 *μ*g/mL) (Invivogen, San Diego, CA) in RPMI-1640 containing 10% FBS for 1 h at 37°C and 5% CO_2_. The cells were then stimulated with FAM20 or *lpxA* at an MOI of 10 and simultaneously stained for caspase-1 activity with FLICA for 2 h. Caspase-1 activity was determined by flow cytometry.

### 2.5. Western Blot Analysis

Primary human neutrophils (1 × 10^6^ cells) were seeded in a 96-well plate and challenged with FAM20, *lpxA* (MOI of 10), or LPS (50 ng/mL) in combination with nigericin (10 *μ*M, used as positive control) for 6 h. Cell supernatants were precipitated with 10% trichloroacetic acid for 1 h on ice and resuspended in 4x Laemmli buffer (Bio-Rad Laboratories, Hercules, CA) followed by boiling for 30 min at 95°C. Cellular fractions were harvested in RIPA buffer supplemented with protease and phosphatase inhibitor cocktail (Thermo Fisher Scientific, Rockford, IL). The cell lysate was homogenized with a sharpened pipette tip. The protein concentrations of the cell fractions were measured with DC protein assay (Bio-Rad Laboratories, Hercules, CA). Equal amounts of cell protein were mixed with Laemmli buffer (Sigma-Aldrich, St. Louis, MO) and boiled for 5 min at 95°C. The cell fraction (20 *μ*g) and the precipitated supernatants were subjected to 4–20% TGX Stain-Free™ SDS gel electrophoresis and transferred to an immunoblot polyvinylidene difluoride (PVDF) membrane (Bio-Rad Laboratories, Hercules, CA). The PVDF membrane was blocked with 3% bovine serum albumin in PBS for 1 h. Caspase-1 was detected using a mouse monoclonal antibody (Adipogen AG, Liestal, Switzerland) against human caspase-1 p45, diluted 1 : 1000. As a loading control, GAPDH was detected with a rabbit polyclonal antibody (Santa Cruz Biotechnology Inc., Dallas, TX) diluted 1 : 10 000. All primary antibodies were incubated overnight. As a secondary horseradish peroxidase- (HRP-) conjugated antibody, a goat polyclonal anti-rabbit or anti-mouse IgG was used (Abcam, Cambridge, UK). The blots were developed using the chemiluminescence western blotting detection reagent Luminata Forte Western HRP (Merck Millipore, Billerica, MA).

### 2.6. Total RNA Extraction and cDNA Synthesis

Total RNA was extracted from isolated neutrophils and THP1 cells using Thermo Scientific GeneJET RNA Purification Kit (Thermo Fisher Scientific, Rockford, IL) according to the manufacturer's instructions. The concentration and purity of the RNA were determined by a NanoDrop ND-1000 spectrophotometer (NanoDrop Technology Inc., Wilmington, DE).

cDNA was synthesized using a reverse transcriptase kit (High Capacity Reverse Transcription Kit, Applied Biosystems, Foster City, CA) from 1 *μ*g of total RNA according to the manufacturer's instructions.

### 2.7. Reverse Transcriptase qPCR Analysis

Gene expression analysis was performed using the TaqMan® Gene expression assay consisting of unlabelled primers and target-specific FAM™ dye-labelled probes, specific for *NLRP3* (HS 00918082_m1), *CASP1* (*pro-caspase-1*; HS00354836_m1), *IL1B* (*pro-IL-1β*; HS01555410_m1), and *GAPDH* (HS02758991_g1) detected in a 7900HT Fast Real-Time PCR System (Applied Biosystems, Foster City, CA). Samples were run as singleplex, and expression of target genes was determined in duplicates and normalized to *GAPDH*.

### 2.8. Enzyme-Linked Immunosorbent Assay (ELISA)

Isolated neutrophils were seeded at 2 × 10^5^ in a 96-well plate and challenged with FAM20 or *lpxA* (MOI of 10), for 6 h with or without preincubation for 1 h with the NLRP3 inhibitor MCC950 in a titrated dose (2 *μ*M) balancing blocking capacity and toxic effects (Avistron Chemistry Services, Cornwall, UK). Isolated neutrophils were stimulated with FAM20, *lpxA*, purified LOS 1 *μ*g/mL, *lpxA* + LOS, or FAM20 + TLR4 signalling blockers (anti hCD14-IgA 500 ng/mL, LPS-RS ultrapure 1 *μ*g/mL, and polymyxin B 10 *μ*g/mL) in RPMI-1640 containing 10% FBS for 6 h at 37°C and 5% CO_2_ with or without preincubation for 1 h with the TLR1/2 agonist Pam3CSK4 (2 *μ*g/mL). Furthermore, wild-type THP1 cells, THP1-defNLRP3, and THP1-defCASP1 cells were challenged with FAM20 for 6 h. TNF, IL-6, and IL-1*β* secretion into culture supernatants were analyzed by the ELISA method using ELISA MAX™ Deluxe kit (BioLegend, San Diego, CA) according to the manufacturer's instructions.

### 2.9. Ethical Considerations

The study was conducted in accordance with the ethical guidelines of declaration of Helsinki and followed the ethical policy at Örebro University Hospital, Sweden. The blood samples were anonymized by the Department of Transfusion Medicine at Örebro University Hospital, thereby preventing data to be traced back to a certain individual. Since the blood was withdrawn at the time for blood donation, no extra harm or risk was put to the donors; the study did not require ethical approval according to paragraph 4 of the Swedish law (2003 : 460) on Ethical Conduct in Human Research.

### 2.10. Statistical Analyses

Intergroup comparisons were performed by a two-tailed paired *t*-test (for normally distributed data) or a two-tailed Wilcoxon matched-pair signed-rank test (for data not normally distributed). The Kruskal-Wallis test was used to compare unmatched (unpaired) not normally distributed data. Differences were considered as significant at *P* ≤ 0.05 (^∗^), *P* ≤ 0.01 (^∗∗^), or *P* ≤ 0.001 (^∗∗∗^), ns = not significant (Prisma 6; GraphPad Software 6.0, San Diego, CA).

## 3. Results

### 3.1. The *lpxA* Mutant Strain of *N. meningitidis* Fails to Stimulate Caspase-1 Activity in Human Neutrophils and Monocytes

**Figure 1:**
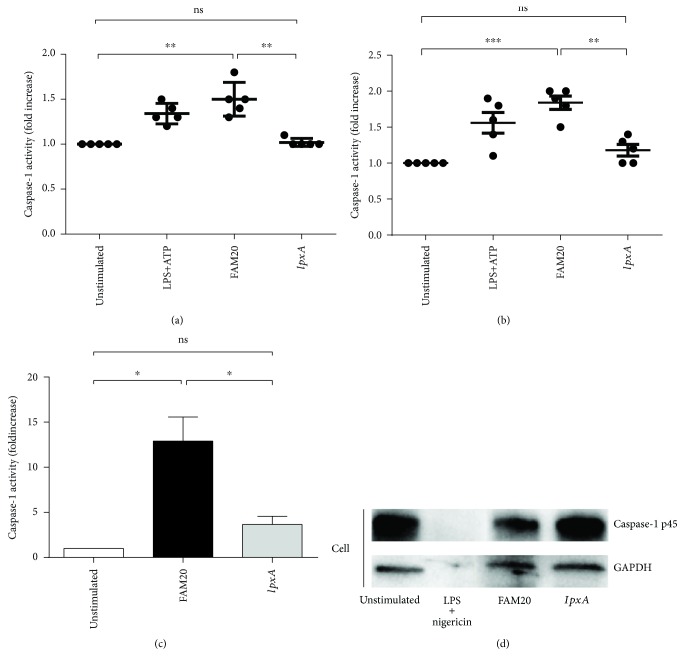
Involvement of LOS in *N. meningitidis*-induced caspase-1 activation in human primary neutrophils and monocytes. Innate immune cells in whole blood (a and b) or isolated human neutrophils (c) were stimulated for 2 h or 6 h, respectively, with the parental wild-type *N. meningitidis* FAM20 or its LOS-deficient mutant strain *IpxA* (MOI of 10). Caspase-1 activity of neutrophils (a) or monocytes (b) was determined as fold increase of median fluorescence intensity (MFI) by flow cytometry or quantified in isolated neutrophils (c) using a caspase-1 fluorometric assay. Bars represent mean ± SD, *n* = 5 (a and b) and *n* = 4 (c), of data normalized to unstimulated controls. Western blot (d) was used to detect caspase-1 in the uncleaved p45 form in primary human neutrophils stimulated with wild-type FAM20, *lpxA* mutant, or LPS (50 ng/mL) in combination with nigericin (10 *μ*M). Results are shown as one representative out of three independent experiments (d).

Since the activated inflammasome recruits pro-caspase-1 and triggers its activation, the level of cleaved caspase-1 was analyzed as a marker of inflammasome activation [25, 29], both in neutrophils and in monocytes in whole blood as well as in isolated neutrophils. The results showed that caspase-1 activity was significantly increased in neutrophils (*p* = 0.0039, Figure 1(a)) and monocytes (*p* = 0.0008, Figure 1(b)) stimulated with the FAM20 strain when compared to unstimulated cells in whole blood. Neutrophils and monocytes stimulated with the mutant *lpxA* strain demonstrated similar low caspase-1 activity as found in unstimulated cells (Figures 1(a) and 1(b)). The same results were obtained using isolated human neutrophils (*p* = 0.02, Figure 1(c)). Caspase-1 activation was confirmed using Western blot showing that the amount of pro-caspase-1 (p45) was reduced in the cells of FAM20-stimulated neutrophils, whereas no reduction of p45 was detected in cells stimulated by *lpxA* (Figure 1(d)).

### 3.2. Stimulation of Innate Immune Cells by *N. meningitidis lpxA* Mutant Strain Results in Lower IL-1*β* Release Compared to Wild-Type FAM20 Strain

**Figure 2:**
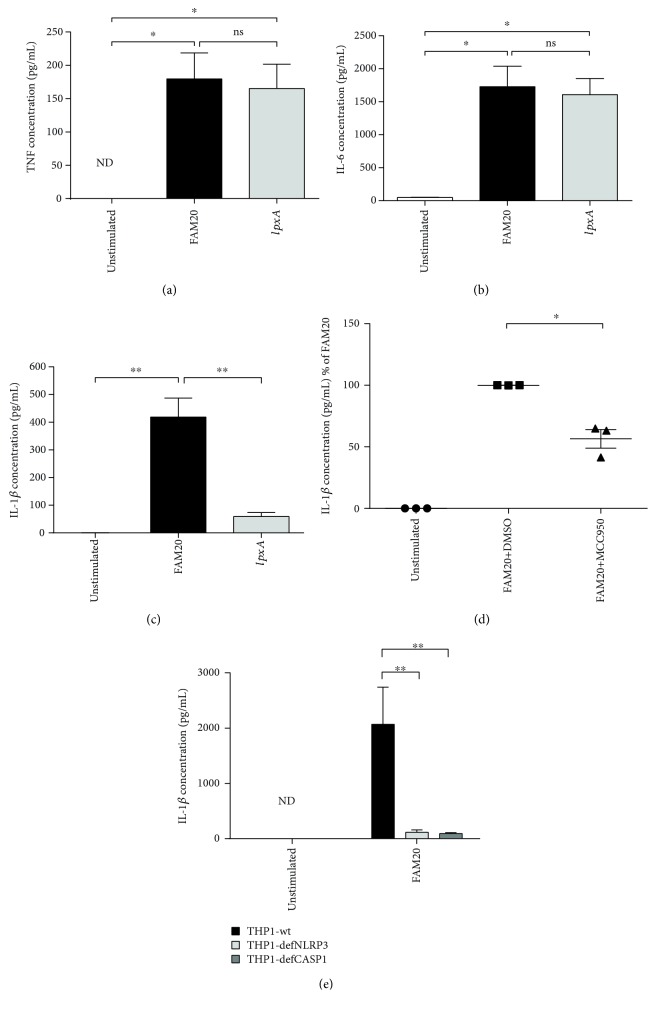
Involvement of LOS in *N. meningitidis*-induced secretion of TNF and IL-6 and NLRP3 inflammasome/caspase-1/IL-1*β* axis-dependent IL-1*β* secretion. Isolated human neutrophils were challenged (MOI of 10) with the parental wild-type *N. meningitidis* FAM20 or its LOS-deficient mutant strain *IpxA* for 6 h after TNF (a), IL-6 (b), IL-1*β* (c) secretion was measured from culture supernatant by ELISA. Bars represent mean ± SEM; *n* = 4. In some experiments, the isolated human neutrophils were preincubated with the NLRP3 inhibitor MCC950 prior to FAM20 stimulation (MOI of 10) (d). THP1-wt, THP1-defNLRP3, and THP1-defCASP1 cells were challenged with FAM20 (MOI of 10) for 6 h (e), and IL-1*β* release was measured from culture supernatant by ELISA. Bars represent mean ± SEM; *n* = 3.

In compliance with previously detected caspase-1 activity (Figure 1), stimulation of innate immune cells with FAM20 resulted in significantly higher amounts of IL-1*β* in the supernatant than did the LOS-deficient *lpxA* mutant (*p* = 0.0083) (Figure 2(c)). On the other hand, FAM20 as well as *lpxA* gave rise to similar increased levels of secreted TNF (Figure 2(a)) and IL-6 (Figure 2(b)) from innate immune cells when compared with unstimulated cells.

### 3.3. NLRP3 Inflammasome Plays a Key Role in the Secretion of IL-1*β* in Innate Immune Cells Stimulated by *N. meningitidis*

To investigate if the LOS-dependent secretion of IL-1*β* is dependent on NLRP3 activation, isolated neutrophils were preincubated with the NLRP3 inhibitor MCC950 prior to stimulation with wild-type FAM20. A significantly reduced level of IL-1*β* was detected in cells treated with MCC950 prior to stimulation with FAM20 (*p* = 0.0291, Figure 2(d)). Moreover, the THP1-defNLRP3 and THP1-defCASP1 cell models were used to investigate the involvement of the NLRP3 inflammasome/caspase-1/IL-1*β* axis in facilitating the secretion of IL-1*β* mediated by *N. meningitidis*. THP1-defCASP1 cells were incapable of activating IL-1*β* (Figure 2(e)), and in line with our data using the NLRP3 inhibitor, secretion of IL-1*β* was significantly reduced in THP1-defNLRP3 cells (*p* = 0.0015, Figure 2(e)) compared with wild-type THP1 cells after being challenged with FAM20—data indicating the involvement of the NLRP3 inflammasome in *N. meningitidis*-induced inflammation.

### 3.4. Both *N. meningitidis* Wild-Type FAM20 Strain and *lpxA* Mutant Strain Induce Gene Expression of *NLRP3* and *IL1B* but Not *CASP1* in Innate Immune Cells

**Figure 3:**
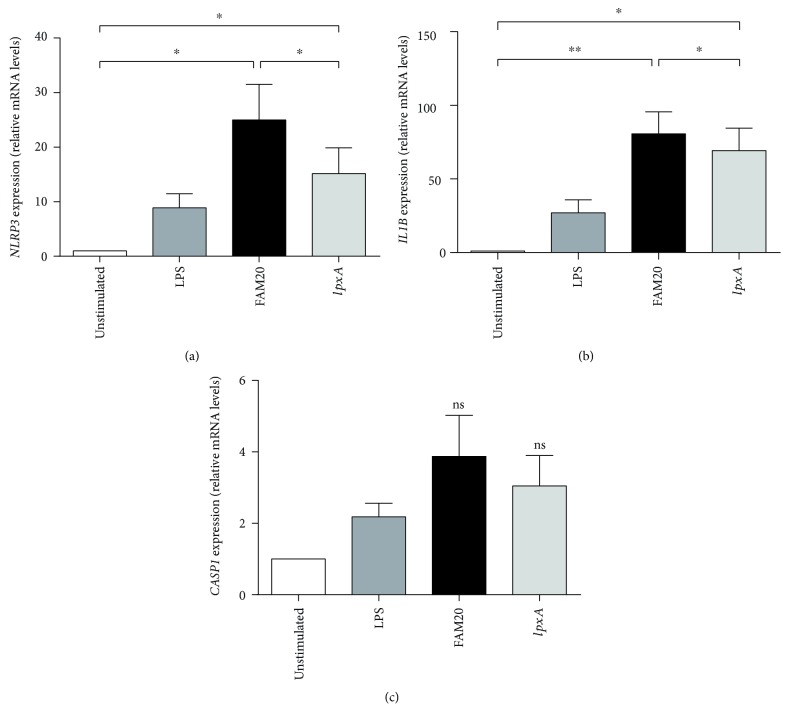
Involvement of LOS in *N. meningitidis*-induced mRNA expression of inflammasome components. Isolated human neutrophils were challenged by the parental wild-type *N. meningitidis* FAM20 or its LOS-deficient mutant strain *IpxA* at MOI 10 or by LPS (50 ng/mL) for 2 h. The mRNA expression of *NLRP3* (a), *IL1B* (b), and *CASP1* (c) was measured by RT-qPCR. Relative gene expression was calculated using the 2^-∆∆C^_T_ method, using *GAPDH* as the reference gene and the unstimulated cells as the calibrator. Bars represent mean ± SEM (*n* = 5).

To investigate the role of LOS in mediating inflammasome priming, the mRNA expression of *NLRP3*, *CASP1* (pro-caspase-1), and *IL1B* (pro-IL-1*β*) was analyzed following bacterial challenge of isolated neutrophils. Stimulation by FAM20 as well as *lpxA* increased the expression of *NLRP3* and *IL1B* in neutrophils, in which FAM20 produced a more pronounced expression in comparison to *lpxA* (Figures 3(a) and 3(b)). In contrast, no increase in the mRNA expression of *CASP1* was observed in neutrophils stimulated with neither FAM20 nor *lpxA* compared to unstimulated cells (Figure 3(c)).

### 3.5. The *N. meningitidis*-Induced mRNA Expression of *NLRP3* and *IL1B*is MyD88-Dependent

**Figure 4:**
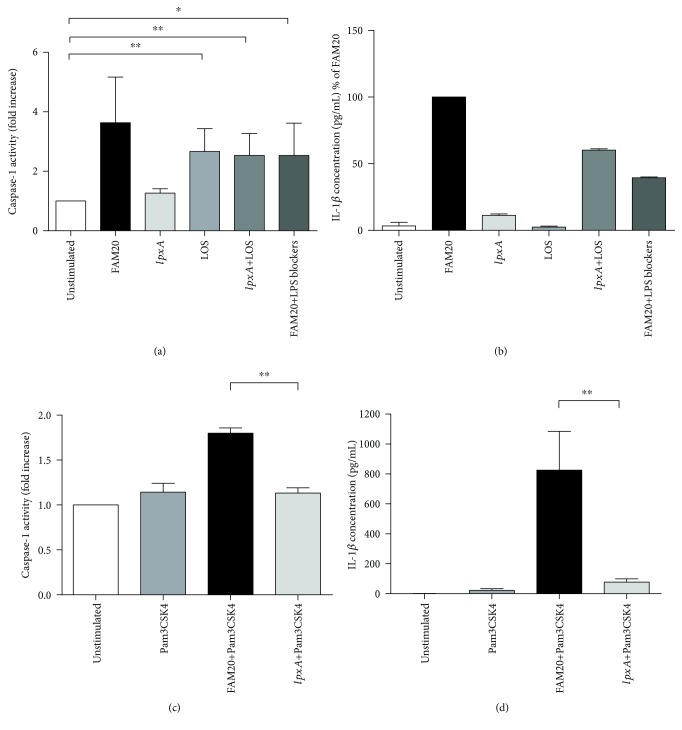
Involvement of LOS in licensing inflammasome activation in innate immune cells. Isolated human neutrophils were stimulated by the parental wild-type *N. meningitidis* FAM20 or its LOS-deficient mutant strain *IpxA* (MOI of 10), by purified LOS alone (1 *μ*g/mL), *lpxA* + LOS, or FAM20 + TLR4 signalling blockers (anti-hCD14-IgA 500 ng/mL, LPS-RS ultrapure 1 *μ*g/mL, and polymyxin B 10 *μ*g/mL) for 2 h and 6 h, respectively (a and b). Isolated neutrophils were primed by preincubation for 1 h with Pam3CSK4 (2 *μ*g/mL) before bacterial stimulation (c and d). Caspase-1 activity of isolated neutrophils (a and c) was determined by flow cytometry and is presented as fold increase in median fluorescence intensity (MFI) normalized to unstimulated cells. IL-1*β* release (b and d) was measured from culture supernatant by ELISA. Bars represent mean ± SEM; *n* = 3.

**Figure 5:**
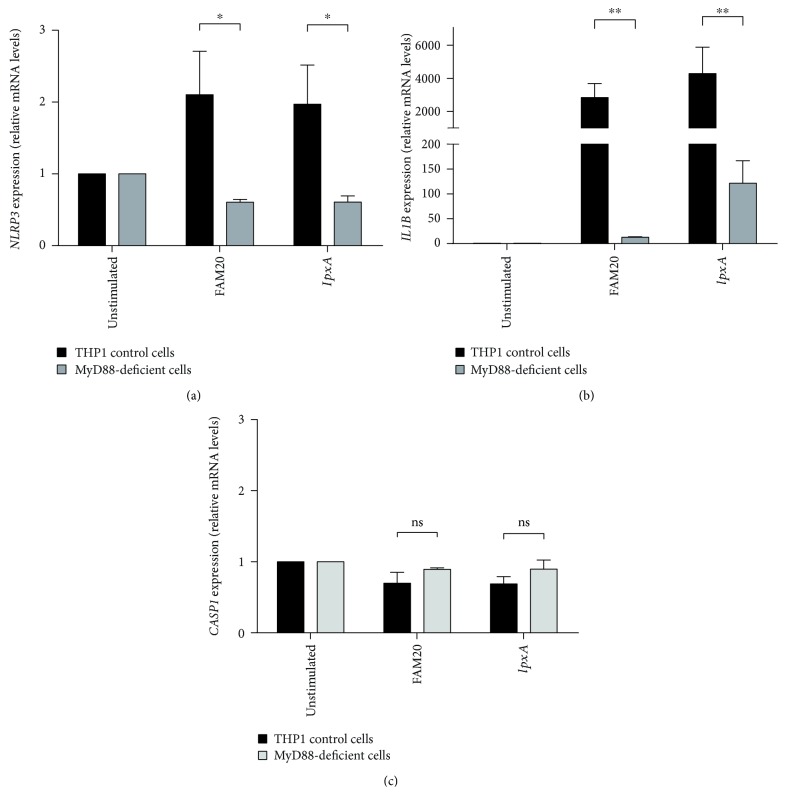
Involvement of MyD88 signalling in *N. meningitidis* priming of NLRP3 inflammasomes. THP1 wild-type control cells and MyD88-deficient THP1 cells were subjected to the parental wild-type *N. meningitidis* FAM20 or its LOS-deficient mutant strain *IpxA* (MOI of 10) for 2 h, after mRNA expression of *NLRP3* (a), *IL1B* (b), and *CASP1* (c) was measured using RT-qPCR. Relative gene expression was calculated with the 2^-∆∆C^_T_ method, using *GAPDH* as the reference gene and the unstimulated cells as the calibrator. Bars represent mean ± SEM; *n* = 3.

LOS is a potent activator of the TLR4/MD2-receptor complex, involving downstream signalling via NF-*κ*B [30]. Our results suggest a role for LOS both in the priming step of the inflammasome (Figure 3) and being crucial for licensing of inflammasome activation (Figures 1 and 4). To determine the involvement of TLR-mediated signalling for priming, we employed THP1 cells deficient in MYD88 and found them unable to respond to bacterial challenge with regards to mRNA expression of *NLRP3*, *IL1B*, and *CASP1* (Figure 5). The priming response in wild-type THP1 cells was in agreement with results from isolated human neutrophils (Figure 3), showing increased mRNA expression of *NLRP3* and *IL1B* in cells stimulated with FAM20 or *lpxA* (Figures 5(a) and 5(b)), whereas neither FAM20 nor *lpxA* was able to increase *CASP1* expression (Figure 5(c)).

### 3.6. LOS of *N. meningitidis* Acts as the Licensing Stimuli for the NLRP3 Inflammasome Activation in Innate Immune Cells

Our data suggest that LOS has a central role in IL-1*β* secretion (Figure 2(c)) and that this is mediated via the NLRP3 inflammasome/caspase-1/IL-1*β* axis (Figures 1, 2(d), and 2(e)). Next, the role of LOS as the licencing step for inflammasome activation was further investigated by stimulating isolated neutrophils with FAM20, *lpxA per se*, LOS *per se*, or by *lpxA* + LOS. In line with our previous data (Figure 1), *lpxA* was unable to induce caspase-1 activity but by adding purified LOS, caspase-1 activity was established (Figure 4(a)). Also, FAM20 was able to increase caspase-1 activity in the presence of TLR4 signalling blockers, but somewhat reduced compared to FAM20 alone (Figure 4(a)). Taken together, these data further support our findings of LOS as a trigger, and not only a primer, of caspase-1 activation. In support of a LOS-dependent caspase-1 activation, *lpxA* + LOS resulted also in a more pronounced secretion of IL-1*β* in comparison to *lpxA per se* or to LOS alone (Figure 4(b)). The role of LOS in licensing of inflammasome activation was further investigated in isolated neutrophils preincubated with TLR1/2 agonist Pam3CSK4 prior to challenge with FAM20 or *lpxA*. These experiments showed a significantly increased caspase-1 activity in neutrophils stimulated by Pam3CSK4 + FAM20, whereas no caspase-1 activity was detected in preprimed cells stimulated by *lpxA* (Figure 4(c)). Similarly, we detected high levels of IL-1*β* in the supernatants of Pam3CSK4 + FAM20-stimulated neutrophils but not in Pam3CSK4 + *lpxA*-stimulated cells (Figure 4(d)).

## 4. Discussion

In septic patients, even low plasma levels of meningococcal LOS (<1 ng/mL) are associated with meningococcal septic shock and death [9, 31] and LOS is a potent inducer of proinflammatory cytokines, including IL-1*β*, TNF, IL-6, and CXCL8, in human and murine macrophages [8, 10, 31] as well as IL-1*β* in human blood *ex vivo* [30]. IL-1*β* maturation into a bioactive molecule and its secretion require the composition of the inflammasome, which includes the activated enzyme caspase-1. To date, the role of inflammasomes in the response to *N. meningitidis* is unknown and the present study investigates the role of LOS in *N. meningitidis*-mediated stimulation of inflammasome activity in human innate immune cells and suggests a role for inflammasome signalling in meningococcal infections. Our data further show that the LOS-induced response is not a general inflammatory response but rather target the NLRP3/caspase-1/IL-1*β* axis.

Crucial for the interpretation of our data is the realization that inflammasome activation is a two-step process [32] with the priming step being TIR/MyD88/NF-*κ*B-dependent production of pro-IL-1*β* and inflammasome components (e.g., NLRP3 and pro-caspase-1) through stimulation by mainly PAMPs, such as LPS, or by certain endogenous stimuli (e.g., IL-1*β* and TNF) [20]. To determine the role of LOS in regulating inflammasome activity, we studied the priming step by analysing the mRNA expression of *NLRP3*, *CASP1*, and *IL1B* in human neutrophils following stimulation by the parental FAM20 serogroup C *N. meningitidis* and its LOS-deficient mutant strain *IpxA*. Murine neutrophils have been found to express *NLRP3*, *IL1B*, and *CASP1* at resting conditions, and mRNA as well as protein levels of NLRP3 are upregulated via NF-*κ*B-mediated signal transduction [21, 22, 33], whereas no such information is available regarding *IL1B* and *CASP1* in human neutrophils [33]. Although stimulation by both FAM20 and *lpxA* resulted in upregulation of *NLRP3* as well as *IL1B* mRNA levels in isolated human neutrophils, FAM20 produced a more pronounced increase. These data not only show that LOS of *N. meningitidis* plays a role in the priming but also demonstrate that other bacterial factors contribute to the priming step. As non-LOS virulence factors of *N. meningitidis*, such as PorB, pili and capsular polysaccharides trigger the NF-*κ*B pathway [31, 34, 35]; they constitute likely candidates for the priming of inflammasome activity detected in cells stimulated with *lpxA*. The lack in upregulated *CASP1* following stimulation is in line with data from murine bone marrow-derived macrophages, where pro-caspase-1 expression is not affected by LPS stimulation [22]. Our data support the inflammasome priming model but specifically add information of this process in human innate immune cells by suggesting that *NLRP3* and *IL1B* are induced by *N. meningitidis*, whereas the expression of *CASP1* is not.

The priming properties of LOS were further investigated by inhibiting its interaction with cells by sequestering LOS with polymyxin B, by blocking of CD14, and by competitive binding to TLR4 by LPS-RS during FAM20 challenge of human neutrophils. Even if the cells were able to produce IL-1*β* to approximately 50% of non-inhibited cells during such conditions, the results indicate additional signalling pathways, other than TLR4, in inflammasome priming. These observations are supported by reports describing that wild-type *N. meningitidis* mediates NF-*κ*B activation via the TLR4/MD2/MyD88-pathway, while LOS-deficient strains contribute to inflammation by non-LOS molecules via activation of TLR2/MyD88 [30, 36], indicating the involvement of both TLR2 and TLR4 pathways in the induction of inflammasome priming. The involvement of the MyD88/NF-*κ*B pathway in the priming process of the inflammasome was therefore explored utilizing MyD88-deficient THP1 cells, and we found a complete MyD88 dependency for *N. meningitidis*-induced inflammasome priming. Taken together, these data imply that the non-LOS virulence factors of *N. meningitidis* act via TLR2 to induce priming in the TLR4-blocking experiments described above—data supported by Guarda and coworkers, who show inflammasome priming via both TLR2, -3, and -4 [22].

Our results also indicate LOS to be crucial for the licensing of inflammasome activation as we found *lpxA* unable to induce a substantial IL-1*β* release even in pre-primed cells. The pre-primed experiment indicates that the lacking capacity to activate the inflammasome enough to induce IL-1*β* production and release is not due to the lower levels of *NLRP3* expression seen in the priming studies but rather that the capacity to license inflammasome activation itself is defective in the *lpxA* bacteria. The lack of IL-1*β* release by the LOS-deficient bacteria can be explained by their inability to trigger caspase-1 activity in human innate immune cells. Our data is supported by Brandtzaeg and co-workers showing that the LOS-deficient *N. meningitidis* strain (H44/76) is incapable of inducing IL-1*β* from immune cells [30]. We further expand on this data and show that the deficiency of *lpxA* to induce IL-1*β* release can be rescued by the addition of LOS—data suggesting the critical role of LOS in licensing of the NLRP3 inflammasome activation. Even though we found that purified LOS *per se* induces caspase-1 activity, it was unable to increase the production and release of IL-1*β*, which can be regarded as a measure of functionality. Consequently, to initiate a response by the NLRP3/caspase-1/IL-1*β* axis in neutrophils, LOS *per se* requires other virulence factors of the meningococcal bacterium, as proven by the experiment in which *lpxA* + purified LOS generate IL-1*β* secretion by induction of priming and licensing of inflammasome activity, respectively. These observations agree with the common notion that the activation and release of IL-1*β* require both a priming (TLR-engagement of LOS) and a licensing trigger (other *Neisseria* factors) for activation as well as release of IL-1*β*. This result is further strengthened by our experiment where the capacity to induce caspase-1 activity and mediate production and release of IL-1*β* of FAM20 is brought down to the levels of *lpxA* in the presence of TLR4 signalling blockers, which inhibit the effects of LOS.

Even if the involvement of other NLRs and inflammasomes cannot be ruled out and may be coparticipants, our data using the NLRP3 inhibitor and NLRP3-deficient cells show a clear role for NLRP3 in *N. meningitidis*-induced IL-1*β* production. In concordance, NLRP3 is the primary NLR required for IL-1*β* secretion of monocytes in response to another bacterium of the *Neisseria* species, i.e., *N. gonorrhoeae* [37]. Not to overlook in this regard, production of proinflammatory cytokines was recently described through a TLR-independent manner with caspase-4/5 involvement giving rise to caspase-1 oligomerization and inflammasome activation in human monocytes [38]. Whether the LOS-induced pathway involves caspase-1 activation only mediated via NLRP3 and/or via the caspase-4/5 as well will be the subject for future studies awaiting the necessary tools. Of interest to the current study is that, whereas murine monocytic cells only receive priming following LPS challenge, both priming and licensing of inflammasome activation are reported in human monocytes [39], supporting our current data that LOS acts both on the priming and on the licensing of inflammasome activation. In addition, our results indicate that LOS of different serogroups does exert a similar activation of the NLRP3/caspase-1/IL-1*β* axis.

In conclusion, our results provide an explanation to the mechanism behind the LOS-mediated cytokine production in human neutrophils by showing that *N. meningitidis* trigger the inflammasome/caspase-1/IL-1*β* axis in human neutrophils and monocytes. LOS exerts a dual function in the process by stimulating the priming of the cell in concert with additional virulence factors but seem to be the central molecule for the licensing of inflammasome activation leading to a fully functional inflammasome. Neutrophils make up 40-60% of the white blood cell count in the blood and therefore provide a potent source of IL-1*β*, the proinflammatory cytokine so tightly connected to severe meningococcal septic disease [40].

## Data Availability

All data used to support the findings of this study are available from the corresponding author upon request.

## Abbreviations

ELISA: Enzyme-linked immunosorbent assay
FLICA: Fluorochrome inhibitor of caspases
GC-agar: Gonococcus agar
IL: Interleukin
LOS: Lipooligosaccharide
LPS: Lipopolysaccharide
LRR: Leucine-rich repeat
MYD88: Myeloid differentiation primary response 88
NF-*κ*B: Nuclear factor-kappa B
NLRs: Nucleotide-binding domain leucine-rich repeat containing receptor
PAMPs: Pathogen-associated molecular patterns
PRRs: Pattern recognition receptors
ROS: Reactive oxygen species
THP1: Monocytic leukemia cell line
THP1-defCASP1: Human monocytes with reduced caspase-1 activity
THP1-defMYD88: Human monocytes with reduced MYD88 activity
THP1-defNLRP3: Human monocytes with reduced NLRP3 activity
TIR: Toll/interleukin-1 receptor homology
TLRs: Toll-like receptors
Tfp: Type IV pili
TNF: Tumor necrosis factor.
